# Circadian rhythm biomarker from wearable device data is related to concurrent antidepressant treatment response

**DOI:** 10.1038/s41746-023-00827-6

**Published:** 2023-04-29

**Authors:** Farzana Z. Ali, Ramin V. Parsey, Shan Lin, Joseph Schwartz, Christine DeLorenzo

**Affiliations:** 1 Department of Biomedical Engineering, Stony Brook University, 100 Nicolls Road, Stony Brook, NY 11794 USA; 2 Department of Psychiatry, Renaissance School of Medicine at Stony Brook University, 100 Nicolls Road, Stony Brook, NY 11794 USA; 3 Department of Psychology, Stony Brook University, 100 Nicolls Road, Stony Brook, NY 11794 USA; 4 Department of Radiology, Stony Brook University, 100 Nicolls Road, Stony Brook, NY 11794 USA; 5 Department of Electrical and Computer Engineering, Stony Brook University, 100 Nicolls Road, Stony Brook, NY 11794 USA; 6 Department of Psychiatry, Columbia University, 1051 Riverside Drive, New York, NY 10032 USA

**Keywords:** Human behaviour, Predictive markers, Biomedical engineering, Applied mathematics, Depression

## Abstract

Major depressive disorder (MDD) is associated with circadian rhythm disruption. Yet, no circadian rhythm biomarkers have been clinically validated for assessing antidepressant response. In this study, 40 participants with MDD provided actigraphy data using wearable devices for one week after initiating antidepressant treatment in a randomized, double-blind, placebo-controlled trial. Their depression severity was calculated pretreatment, after one week and eight weeks of treatment. This study assesses the relationship between parametric and nonparametric measures of circadian rhythm and change in depression. Results show significant association between a lower circadian quotient (reflecting less robust rhythmicity) and improvement in depression from baseline following first week of treatment (estimate = 0.11, F = 7.01, *P* = 0.01). There is insufficient evidence of an association between circadian rhythm measures acquired during the first week of treatment and outcomes after eight weeks of treatment. Despite this lack of association with future treatment outcome, this scalable, cost-effective biomarker may be useful for timely mental health care through remote monitoring of real-time changes in current depression.

## Introduction

Major depressive disorder (MDD) is one of the most common mood disorders (https://www.nimh.nih.gov/health/statistics/major-depression.shtml) and a research area of high priority in the United States^1^, where approximately 21 million adults had at least one major depressive episode in a year^2^. Globally, an estimated 280 million^3^ individuals are affected by this medical condition considered to be the most disabling (http://www.who.int/mediacentre/factsheets/fs369/en/). Despite the past two decades of policy changes aimed at increasing coverage and reducing stigma associated with mental health care, numbers of people seeking and receiving treatment for depression are disproportionately low in underserved communities^4^. This is alarming since treatment outcome from antidepressant is affected by the duration of depression^5,6^ and time to start treatment^7,8^. Establishing objective biomarkers of treatment efficacy for remote monitoring of depression can address this issue by facilitating timely intervention. This would reduce patient suffering and healthcare costs^9,10^, as conventional methods of depression assessment can be time-consuming and resource-intensive and require a trained clinician^11^. This would particularly benefit those who lack timely access to affordable, high-quality mental health care^4^.

Gross motor activity and body movements are altered in MDD^12^, and a significant feature of MDD is psychomotor retardation^12,13^. Most common complaints among MDD patients include somatic symptoms such as lack of energy, fatigue, general aches and pains that can adversely affect motor activity^14,15^. Such somatic symptoms are present in up to two thirds of depressed patients, but can go undetected in current clinical practice^16–18^. The questionnaire used for assessing depression focused on current complaints can lead to underreporting, that can be addressed by using objective measures related to somatic characteristics from wearable monitors^16^.

Depressed patients on naturalistic treatment (both in and out patient) for one week showed a positive association between motor activity and improvement of depression^19^. Several smaller pilot studies have reported such relationship between motor activity and antidepressant response^13,20,21^. Therefore, an automated, objective, and affordable way to assess depression may be through monitoring motor activity which is likely to be lower in depression^22–25^, and increase with depression improvement^22,26,27^.

Actigraphy allows objective and longitudinal assessment of motor activity from the participants’ living environment with minimal discomfort and effort^28^. Even though actigraphy has been used to evaluate sleep-wake changes for nearly half a century, a more modern and promising application in recent years involves its automated measures of circadian motor activity rhythms with date-time stamps^29^. Actigraphy data is obtained using a compact, lightweight, and affordable device with long-term data storage capability that only requires wearing a wrist/waist band with minor intervention (periodic charging)^28^. With the growing popularity of wearable technology, the use of actigraphy devices with valid measures of motor activity has become widespread and the data have become easily accessible^30^.

A correlation between motor activity and depression severity has been reported in studies using actigraphy^11,31^. However, to our knowledge, no study to date has used actigraphy early in treatment to assess initial antidepressant response to conventional MDD treatments. Assessing response in the first week of treatment may be especially important since the initial effect of antidepressants may occur by the end of the first week of treatment^32^. Specifically, it has been observed that patients who exhibit early partial symptomatic improvement are more likely to ultimately respond^33^, and early improvement can be a reliable indicator for an eventual antidepressant response^33,34^.

By fitting a cosine wave to the actigraphy data, parametric measures, including the midline estimating statistic of rhythm (MESOR, average activity level) and amplitude, that may be decreased in MDD^35^, can be extracted. The normalized value of amplitude (normalized by MESOR) is called circadian quotient (CQ). CQ reflects robustness of the circadian rhythm, and may be inversely related to depression severity^36,37^. Another parametric measure is acrophase (time of peak activity) which may be delayed in MDD^26,37^. Baseline and concurrent measures of acrophase have been shown to be related to response after infusion with the fast-acting antidepressant, ketamine^38^. One major limitation of these parametric measures is the assumption of cosine wave pattern of the time series actigraphy data, even though the activity data may not always resemble such a pattern^39^.

This issue can be addressed using nonparametric methods that do not require this assumption. Two such measures are daytime and nighttime activity. The resolution of increased gross motor activity in MDD patients may be more apparent during nighttime hours^12^. Intensified daytime movements following four weeks of treatment have shown correlation with improvement in depression severity^13^. During the first week of treatment in a previous study, the quantity of daytime motor activity was slightly lower and nighttime motor activity was significantly higher in those who improved compared to those who did not improve after four weeks of treatment^40^. Further, MDD patients may have altered rest-activity cycles^41,42^. Nonparametric measures, such as the intradaily variability (IV), which quantifies the fragmentation in rest-activity cycle and interdaily stability (IS), which quantifies the consistency of the activity patterns over multiple days^43^, have shown an association with depression severity^44^.

The purpose of this study was to identify digital biomarkers of motor activity, that have been previously implicated in depression, for assessing improvement in depression with antidepressant treatment. These biomarkers were acquired from actigraphy data (collected using wearable devices) during the first week of antidepressant treatment in participants with MDD and extracted using the aforementioned parametric and nonparametric measures. The overall goal was to assess associations of the circadian rhythm biomarkers (continuous variables) with change in depression after: (1) the first week of treatment (while the actigraphy device was being worn) assessed as change in depression severity (continuous variable), and (2) eight weeks of treatment assessed as: (i) change in depression severity (continuous variable), and (ii) remission status at week eight (binary variable). The primary hypothesis is that improvement in depressive symptoms after the first week and eight weeks of antidepressant treatment will be related to the first week of actigraphy data in participants with MDD.

Actigraphy analysis in this study shows that less robust rhythmicity (a lower CQ) in the first week of treatment is associated with improvement in depression over that first week. There is insufficient evidence to establish any relationship between the circadian rhythm measures acquired during week one and depression outcome after eight weeks of treatment. This study should be replicated on a larger, more diverse sample, with the following refinements of the study design: collecting actigraphy for the full eight weeks and weekly assessment of depression. Such activity-based biomarkers of antidepressant efficacy can provide opportunities for personalized, noncontact, cost-effective mental health care through real-time, unobtrusive monitoring of current depression and characterization of circadian rhythm during antidepressant treatment.

## Results

### Study cohort

The study included actigraphy data collected between 07/05/2017 and 03/13/2020 from 40 participants with a minimum of 4 days (≥5760 min) of actigraphy data (without any missing data). The study sample had an age range of 18 to 64 years, including 28 (70%) females (Table 1). Overall, 16 (40%) participants remitted (remitters, free of depression) after eight weeks of treatment. There was no significant difference between non-remitters (not free of depression) and remitters in the study sample in terms of treatment assignment, sex, or age at α = 0.05 (Table 1). The pretreatment values of depression severity (Week0-HDRS), measured using the Hamilton Depression Rating Scale (HDRS) before treatment, were higher in non-remitters than the remitters (Table 1). The participants’ demographics and clinical characteristics stratified by treatment status (SSRI vs. placebo) did not show any significant difference (Supplementary Table 1).

**Table 1:** Participants’ demographics and clinical characteristics, stratified by remission status.

Variables	Remission Status								
	Non-Remitters (*N* = 24, 60%)			Remitters (*N* = 16, 40%)			Fisher’s exact test	*t*-test (DF = 38)	MW-*U* test
	*N* (%)	Mean (SD)	Median (IQR)	*N* (%)	Mean (SD)	Median (IQR)	(*P*)	*t* (*P*)	*z* (*P*)
Treatment assignment									
SSRI (vs. Placebo)	16 (67)			6 (38)			0.11		
Demographic									
Sex: F (vs. M)	17 (71)			11 (69)			1.00		
Age (years)		30.24 (14.20)	24.30 (10.80)		32.18 (17.44)	22.95 (24.65)		−0.39 (0.70)	0.11 (0.91)
Clinical									
Week0-HDRS		19.33 (4.94)	18.00 (5.00)		15.56 (3.85)	15.50 (4.50)		**2.57** (0.01*)	**2.30** (0.02*)
Week1-HDRS		15.92 (3.88)	15.50 (5.50)		13.50 (3.74)	14 (5)		1.96 (0.06)	1.79 (0.07)
Week8-HDRS		14.42 (5.18)	14.00 (6.50)		4.53 (2.19)	4.50 (3.50)		**7.19** (<0.01*)	**5.32** (<0.01*)
ΔHDRS1		−0.20 (0.20)	−0.19 (0.28)		−0.15 (0.29)	−0.22 (0.27)		−0.61 (0.54)	−0.07 (0.94)
ΔHDRS8		−0.32 (0.30)	−0.24 (0.41)		−1.39 (0.75)	−1.13 (0.48)		**6.30** (<0.01*)	**5.03** (<0.01*)

Non-Remitters: Week8-HDRS > 7. Remitters: Week8-HDRS ≤ 7.

*HDRS* Hamilton depression rating scale, *Week0-HDRS* Hamilton depression rating scale score at baseline, *Week1-HDRS* Hamilton depression rating scale score after first week of treatment, *Week8-HDRS* Hamilton depression rating scale score after eight weeks of treatment, *ΔHDRS1*: change in depression severity after first week of treatment, ln(Week1-HDRS/ Week0-HDRS), with a higher value indicating more depression symptoms and a lower value indicating improvement in depression. *ΔHDRS8*: change in depression severity after eight weeks of treatment, ln(Week8-HDRS/ Week0-HDRS), with a higher value indicating more depression symptoms and a lower value indicating improvement in depression. *N* sample size. *DF* degrees of freedom = 38 for all the variables in the *t*-test. *MW-U test* Mann–Whitney *U* test, *SD* standard deviation, *IQR* interquartile range (75th percentile – 25th percentile), *SSRI* selective serotonin reuptake inhibitor, *F* female, *M* male.

The *P*-values that are statistically significant are indicated in bold for *P < 0.05 at α = 0.05.

### Statistical analysis

All analysis in this study included all participants (*n* = 40) without any missing data. The Kolmogorov-Smirnov test showed that the distribution of the log-transformed values for change in depression after one week [ΔHDRS1 = ln(Week1-HDRS/Week0-HDRS) = ln(Week1-HDRS) – ln(Week0-HDRS)] and after eight weeks [ΔHDRS8 = ln(Week8-HDRS/Week0-HDRS) = ln(Week8-HDRS) – ln(Week0-HDRS)] of treatment followed normal distribution (D = 0.10, *P* > 0.15). No significant difference in parametric or nonparametric measures were found between non-remitters and remitters using two-tailed t-tests or Mann-Whitney U (MW-U a.k.a. Wilcoxon Rank Sum) test at α = 0.05 (Table 2).

**Table 2:** Participants’ circadian rhythm measures, collected during first week of treatment, stratified by remission status and their relation to change in depression.

Variables	Remission Status							
	Non-Remitters (*N* = 24, 60%)		Remitters (*N* = 16, 40%)		*t*-test (DF = 38)	MW-*U* test	Δ HDRS1	Δ HDRS8
	Mean (SD)	Median (IQR)	Mean (SD)	Median (IQR)	*t* (*P*)	*t* (*P*)	r_s_ (*P*)	
Parametric Circadian Rhythm Measures								
MESOR (counts)	195.59 (91.80)	221.52 (111.76)	219.31 (102.37)	229.29 (73.38)	−0.76 (0.45)	−0.80 (0.42)	−0.10 (0.53)	−0.06 (0.73)
Amplitude (activity counts/minute)	165.45 (85.43)	172.89 (112.61)	156.99 (83.97)	182.08 (129.31)	0.31 (0.76)	0.14 (0.89)	0.11 (0.51)	0.09 (0.59)
CQ	0.83 (0.14)	0.86 (0.15)	0.75 (0.22)	0.78 (0.37)	1.41 (0.17)	0.64 (0.53)	0.38 (0.02*)	0.12 (0.44)
Acrophase	−4.08 (0.99)	−4.29 (0.77)	−4.16 (0.68)	−4.09 (0.99)	0.28 (0.78)	−0.36 (0.72)	−0.03 (0.84)	−0.04 (0.81)
Nonparametric Circadian Rhythm Measures								
M10	311.38 (156.06)	307.62 (193.26)	333.07 (159.56)	363.76 (171.38)	−0.43 (0.67)	−0.58 (0.56)	−0.02 (0.90)	−0.05 (0.75)
L5	38.28 (33.07)	26.45 (63.94)	64.00 (73.28)	39.95 (33.10)	−1.51 (0.14)	−1.05 (0.29)	−0.24 (0.14)	−0.21 (0.19)
RA	0.74 (0.20)	0.79 (0.29)	0.72 (0.24)	0.82 (0.27)	0.32 (0.75)	0.21 (0.84)	0.29 (0.07)	0.18 (0.26)
IV	1.05 (0.30)	1.02 (0.33)	1.10 (0.28)	1.13 (0.28)	−0.53 (0.60)	−0.76 (0.45)	−0.11 (0.49)	−0.20 (0.21)
IS	0.35 (0.15)	0.36 (0.18)	0.32 (0.12)	0.34 (0.10)	0.64 (0.53)	0.35 (0.73)	0.20 (0.21)	0.03 (0.86)

Non-Remitters: Week8-HDRS > 7. Remitters: Week8-HDRS ≤ 7.

*HDRS* Hamilton depression rating scale, *Week0-HDRS* Hamilton depression rating scale score at baseline, *Week1-HDRS* Hamilton depression rating scale score after first week of treatment, *Week8-HDRS* Hamilton depression rating scale score after eight weeks of treatment, *ΔHDRS1*: change in depression severity after first week of treatment, ln(Week1-HDRS/ Week0-HDRS), with a higher value indicating more depression symptoms and a lower value indicating improvement in depression. *ΔHDRS8*: change in depression severity after eight weeks of treatment, ln(Week8-HDRS/ Week0-HDRS), with a higher value indicating more depression symptoms and a lower value indicating improvement in depression. *N* sample size, *DF* degrees of freedom = 38 for all the variables in the *t*-test. *MW-U test* Mann–Whitney *U* test, *SD* standard deviation, *IQR* interquartile range (75th percentile – 25th percentile). *r*_*s*_ Spearman’s correlation coefficient, which was used to assess correlation between each of the parametric and nonparametric measures extracted from the first week of treatment and the change in depression after one week (ΔHDRS1) and eight weeks of treatment (ΔHDRS8). *MESOR* midline estimating statistic of rhythm, indicates the average activity level. *Amplitude*: difference between the highest point and average value of the circadian rhythm. *CQ* Circadian Quotient (amplitude/MESOR), which reflects robustness of the circadian rhythm. *Acrophase*: time of peak activity. *M10*: average activity count (min^−1^) during the most active 10 consecutive hours. *L5*: average activity count (min^−1^) during the least active 5 consecutive hours. *RA* relative amplitude, reflecting the difference between M10 and L5 activity. *IV* intradaily variability, estimates the circadian rhythm fragmentation by taking into account the extent and frequency of rest-activity transitions every hour (range: 0–2), with a higher value indicating a more fragmented rest-activity rhythm due to daytime rest/nappings and/or nighttime arousals. *IS* interdaily stability, estimates the consistency/stability of activity patterns over multiple days and its synchronization to the 24-hour light-dark cycle (range: 0–1), with a higher value indicating stable rhythm with good synchronization.

The *P*-values that are statistically significant are indicated in bold for *P < 0.05 at α = 0.05.

### Relationship of week 1 actigraphy measures with change in depression at week 1 and week 8

Of all the parametric and nonparametric measures, circadian quotient (CQ) was the only measure significantly correlated (r_s_ = 0.38, *P* = 0.02) with change in depression severity in the first week of treatment (ΔHDRS1, Table 2). This relationship remained significant even after removing two outliers (green box in Supplementary Fig. 3). No significant correlation was seen between any parametric or nonparametric measure and change in depression severity at week 8.

Using repeated measures analysis of variance (RM-ANOVA) with linear mixed model showed that there was significant association between the standardized value of circadian quotient (CQ) and change in depression from baseline following first week of treatment (estimate = 0.11, t = 2.65, F = 7.01, *P* = 0.01, Table 3). The model had 33 degrees of freedom for all the predictor variables (added as fixed effects). The results showed that for 1 standard deviation (SD) increase in an individual’s CQ, their depressive symptoms after one week of treatment is estimated to be greater (higher depression) by 0.11 point. If individual A was 1 SD above individual B on CQ, then the increase in A’s depressive symptoms is estimated to be 0.11 point greater (higher depression) than the increase in B’s depressive symptoms. Alternatively, the decrease in A’s depressive symptoms (improvement in depression) would be 0.11 point less than the decrease in B’s depressive symptoms. Repeated measures analysis of variance (RM-ANOVA) using linear mixed modeling did not show any statistically significant association between the parametric or nonparametric measures from the first week of treatment and change in depression after eight weeks of treatment.

**Table 3:** Results of the repeated measures analysis of variance with linear mixed model to assess the relationship between parametric circadian rhythm measures, collected during first week of treatment, and change in depression after that first week of treatment.

Dependent Variable: ΔHDRS1 (*N* = 40)				
Effects (DF = 33)	Estimate	Standard Error	F	*P*
SSRI (vs. Placebo)	0.17	0.09	3.48	0.07
Sex: F (vs. M)	−0.16	0.11	2.00	0.17
Age (years)	−0.01	0.03	0.05	0.82
Age_sq	0.00	0.00	0.05	0.82
CQ_std	0.07	0.05	1.95	0.17
Acrophase_std	0.05	0.05	0.98	0.33
Week	−0.44	0.39	1.28	0.27
SSRI*Week	−0.14	0.07	3.93	0.06
Female*Week	−0.07	0.09	0.63	0.43
Age*Week	0.02	0.02	1.12	0.30
Age_sq*Week	−0.00	0.00	1.00	0.32
CQ_std*Week	**0.11**	**0.04**	**7.01**	0.01*
Acrophase_std*Week	−0.01	0.04	0.04	0.85

*ΔHDRS1*: change in depression severity after first week of treatment, ln(Week1-HDRS/ Week0-HDRS), with a higher value indicating more depression symptoms and a lower value indicating improvement in depression. *HDRS* Hamilton depression rating scale. *Week1-HDRS*: Hamilton Depression Rating Scale score after first week of treatment. *Week0-HDRS*: Hamilton Depression Rating Scale score at baseline. *N*: sample size. *DF*: degrees of freedom = 33 for all the predictors in the analysis. *SSRI*: selective serotonin reuptake inhibitor. *F* Female. *M* Male. *CQ_std*: standardized values of circadian quotient (CQ = amplitude/MESOR), which reflects robustness of the circadian rhythm. *Acrophase_std*: standardized values of acrophase reflecting the time of peak activity. *Week:* treatment week (Week 0 vs. Week 1, repeated measure). The *P*-values that are statistically significant are indicated in bold for *P < 0.05 at α = 0.05.

### Prediction of remitters and non-remitters

Multivariate logistic regression models for predicting remission status (remitters vs. non-remitters), with control variables of treatment assignment (SSRI vs. placebo), sex (female vs. male), age and squared value of age, did not show any significant relationship between the predicted odds of remitting after eight weeks of treatment and either the parametric or nonparametric measures in two separate models. Yet, the model with the parametric measures and covariates was able to predict 15 remitters correctly out of 16 remitters in the sample. This logistic regression model with the positive outcome threshold of 0.25 predicted remission status (remitters vs. non-remitters) after eight weeks of treatment with 93.75% sensitivity, 50.00% specificity (67.50% weighted accuracy), 55.56% positive predictive value, and 92.31% negative predictive value.

Using only the statistically significant predictive variable from the RM-ANOVA model for the first week of treatment, CQ, a logistic regression model with the positive outcome threshold of 0.38 predicted remission status (remitters vs. non-remitters) after eight weeks of treatment with 62.50% sensitivity, 62.50% specificity (62.50% weighted accuracy), 52.63% positive predictive value, and 71.43% negative predictive value.

## Discussion

To the best of our knowledge, no previous study has characterized nonparametric and parametric measures of motor activity in participants with MDD early in antidepressant treatment. In addition to such characterization, this study investigated potential role of these circadian rhythm measures in assessing antidepressant treatment response in a placebo-controlled randomized trial (Advancing Personalized Antidepressant Treatment Using PET/MRI, NCT02623205). The focus on acigraphy measures collected during the first week of treatment is because the clinically beneficial effect of antidepressants may occur by the end of the first week of treatment^32^. The covariates in this study include age, sex and treatment type. Of all the measures, circadian quotient (CQ), reflecting rhythm robustness, might be the most robust, since it provides the amplitude normalized by average motor activity (MESOR), and thereby takes into account both daytime and nighttime motor activity. CQ has been previously shown to have a negative correlation with depression severity in people with chronic heart failure^36^, but a positive correlation with depression severity during peripartum period^45^. Little was known about its characteristics in participants with MDD who are undergoing treatment that may affect their rhythmicity.

In this study, a lower CQ in the first week of treatment was correlated with improvement in depression over that first week. This association remains significant after controlling for age, sex and treatment assignment, suggesting that this association is independent of those control variables related to depression. Even though the final remission status was not associated with the first week of actigraphy data in this study with limited power and small sample size, CQ could still be an important factor in future models for assessing final antidepressant response in conjunction with other variables related to circadian rhythm (e.g. sleep)^46^, that are already available from actigraphy.

The association between motor activity and depression severity reported in earlier actigraphy studies could not be reproduced in this study^11,31^. A previous actigraphy study was able to find association of motor activity data with changes in symptoms across 2 weeks, but that study included MDD and bipolar disorder (BD) to increase power, not exclusively MDD as our study^11^. That study used publicly available data without any information on previous medication, that may have affected the study outcome. Five of their patients’ movement might have been restricted for residing in an inpatient facility during actigraphy. Moreover, their assessment contains data from two weeks, whereas this study assessed the relationship between the first week of motor data and depression over that first week or depression over the eighth week of treatment.

MESOR was previously found to be lower in responders, compared to nonresponders, during the first day of ketamine treatment^47^. The fast-acting antidepressant effect of electroconvulsive therapy and ketamine as compared to SSRI and the different type of depression across studies may lead to this type of discrepant findings^48^. The acrophase was not affected by treatment (with electroconvulsive therapy) in a previous actigraphy study in patients with treatment-resistant MDD, where depression was assessed with the 17-item Hamilton Depression Rating Scale used in the current study^31^. This open, non-randomized study had 10 women and 5 men (higher ratio of females compared to the current study) with an average age of 47.9 which is higher than the current study population. Such older population are more likely to respond to ECT^49^. MDD patients have displayed activity rhythm advancement (lower acrophase) one day after infusion with the fast-acting antidepressant, ketamine, which reverted to baseline after three days^38^. The mean age in the study was 42.6 with 57% female. The study included both MDD and BD patients with treatment resistant depression. The study participants were medication free for 2–5 weeks before initiating treatment, but some were on mood stabilizers for BD. The wearable monitor was worn for 2–3 days before and three days after the ketamine infusion. A blunted amplitude was detected in these responders three days after treatment^38^. Amplitude was higher in responders, compared to nonresponders, during the first day of ketamine treatment in another study focused on individuals with treatment-resistant depression^47^. These ketamine studies included both MDD and BD with some receiving mood stabilizers that can affect the findings. Moreover, depression was assessed using Montgomery-Asberg Depression Rating Scale (MADRS) as opposed to HDRS in the ketamine studies. In treatment-resistant depressed patients, ECT resulted in increases of circadian amplitude in remitters, but not non-remitters, which could not be replicated in the current study^31^. The current study did not show any difference in amplitude between remitters and non-remitters during first week of treatment that may lack the rapid response expected from ECT^49^ and ketamine^47^. The ECT sessions averaged between 7.8^49^–9.8^31^ and ketamine was administered as a single dose in those previous studies^38,47^.

Clinical improvement in depressive symptoms after four weeks of treatment in patients with MDD was previously related to daytime, but not nighttime motor activity from the first week of treatment assessed via actigraphy^40^. Daytime motor activity was slightly lower and nighttime motor activity was significantly higher during the first week of treatment in patients with depression who improved (≥50% on HDRS) compared to those who did not improve after four weeks of treatment^40^. Correspondingly, the mean relative amplitude (RA) in our study reflecting the difference between daytime and nighttime activity was lower in the remitters compared to non-remitters, but this finding did not reach statistical significance. In this study, no difference was observed between the remitters and non-remitters in terms of daytime or nighttime activity, and no correlation was found between the first week of daytime and nighttime motor activity data and the clinical improvement after eight weeks of treatment.

To recapitulate, this study did not replicate some of the findings from previous actigraphy studies mainly due to the following differences. (1) Diagnosis: majority of the previous studies included patients with MDD and BD. (2) Mean age: the mean age of the previous studies was much older than the current study cohort. (3) Length of treatment: the length of treatment was mostly shorter compared to the current study. (4) Treatment type: one study used mood stabilizers, while another used antipsychotic drugs during antidepressant treatment, that can affect motor activity.

The treatment assignment of SSRI vs. placebo (binary variable: 0. Placebo, 1. SSRI) was a fixed effect in the repeated measures analysis of variance (RM-ANOVA) using linear mixed model and a covariate in the logistic regression models in the study for analyzing association between circadian rhythm measures and change in depression. The findings partially validated the same response rate expected for placebo and SSRI in mild to moderate depression^50,51^. Prior literature suggests that with treatment, improvement in depression is likely associated with placebo and spontaneous recovery approximately half the time, while the rest may be attributable to active intervention^52,53^. No difference has been observed in treatment-induced neurobiological changes from antidepressants versus placebo^54^. The ECT studies with treatment resistant depressed patients did not have a placebo group (for not expecting a large bias due to placebo effect)^31,49^. One of the aforementioned ketamine studies had a placebo group, but no significant differences were found between MESOR, amplitude or acrophase due to treatment with ketamine or placebo one day before the circadian rhythm measurements^47^. Relatedly, assignment of SSRI or placebo did not affect the final outcome of remitters or non-remitters in the study. More importantly, to our knowledge, no other study has shown a similar lack of difference between SSRI and placebo effects on the characteristics of circadian rhythm measures in MDD patients yet.

A major limitation of this study is the lack of power that can be improved in future by replicating this study in larger, more diverse samples. The study design can be improved by collecting actigraphy for the full eight weeks of treatment and assessing the relationship between circadian rhythm measures and change in depression for each week of treatment. Furthermore, machine learning techniques may help recognize patterns in current data not apparent through conventional statistical analysis by using all parametric and nonparametric measures in a single model with appropriate management of overfitting through hyperparameter optimization^55^. Such a model can be expanded to include other demographics and measures of actigraphy time series data (e.g., energy expenditure, sleep duration, etc.) and activity related circadian genes^56^ as well as clinical notes, lab values, and medical imaging^46,57^ for a comprehensive clinical decision support tool. Continuous actigraphy data can be used to identify changes in circadian motor activity over time, by quantifying patterns in sequential time series data, which is also useful for forecasting^58^. Further, the results may be applicable to other lines of antidepressant treatment, such as cognitive behavioral therapy, transcranial magnetic stimulation, etc. Therefore, such lines of research may create new opportunities and directions at the frontiers of wearable technology.

As an added benefit of such a technique, one of the goals of WHO’s Mental Health Gap Action Programme (mhGAP) is to increase services for mental health care by those who are not specialists in mental health (https://www.who.int/news-room/fact-sheets/detail/depression). Since CQ appears to be a biomarker of concurrent depression severity during treatment, using this biomarker instead of subjective reports could facilitate automated treatment monitoring. Furthermore, such circadian rhythm biomarkers can be continuously recorded in an affordable manner without expert intervention, unlike subjective reports. This can support personalized patient care and empower and engage patients in their care, through accelerated feedback. This monitoring of treatment would be particularly beneficial for underserved communities, including the elderly, low-income populations, and residents in rural areas, who have limited access to mental health care, despite the need for psychiatric treatment^59^.

The relationship between mental health and motor activity has been researched before, however, most of the studies do not provide validation of depression diagnosis from a clinician. In contrast, participants in this study were screened, diagnosed, and treated by a trained clinical team including psychiatrists, a psychiatric nurse practitioner, and psychologists with combined decades of experience in psychological assessment. All the study participants were medication free for at least three weeks before initiation of treatment that was useful for minimizing the confounding effects of prior psychotropic medications. Moreover, the current study design consisted of a comprehensive data analysis plan with a priori designation of the primary outcome of remission, based on the ideal target of antidepressant treatment^60^. The processing techniques for the actigraphy data were based on prior literature. A notable strength of the current study is the younger sample that is more informative for a larger group of adults with depression^61^. The largest meta-analysis of depression studies had an average age of 44 years^62^. An individual patient data meta-analysis on the effect of age on depression treatment outcomes had an average age around 42^63^. The mean age in this study sample is 31 with a median age of 23.6, which reflects the large university population near the research institution. This is an important cohort to study as adults ages 18–29 are most likely to experience depression (https://www.cdc.gov/nchs/products/databriefs/db379.htm). However, rather than the younger participants in this study, nonparametric measures are more useful for older adults who are likely to have less pronounced (frail) circadian rhythm, since the nonparametric measures are not based on assumptions about the robustnest of the circadian rhythm^36^. This ancillary actigraphy study used data from the pre-registered protocol for imaging study, which may have affected the sample size and power. Participants in this study had actigraphy data ranging from 4–7 days during their first week of treatment. This variability in the length of days of actigraphy data in the study sample makes it more generalizable to real life population. The current study design closely resembles the expectations in real life scenarios where individuals may not be adherent to wearing their activity monitoring watches all day for seven consecutive days. The current study sample consisted of participants with variable medication start dates, and during their treatment, activity may have been externally influenced depending on the season and availability of sunlight^64^. Yet, the findings from this study provide a framework on which future research can be built to effectively investigate the relationship between circadian rhythm characteristics and antidepressant outcomes in patients with MDD, and inform researchers and clinicians about the value of these potential biomarkers in depression.

The purpose of this study was to identify digital circadian rhythm biomarkers using actigraphy data from the first week of antidepressant treatment that can assess antidepressant response over the time the actigraphy device is worn and in the future. The parametric and nonparametric measures in the study did not show any relationship with change in depression after eight weeks of treatment. However, circadian quotient, a parametric measure assessing circadian rhythm robustness, has shown utility for assessing current depression during treatment, which could be particularly beneficial for real-time, unobstructive, objective monitoring of antidepressant efficacy and depression improvement remotely in individuals with MDD who lack proper access to mental health care.

## Methods

### Study cohort

**Fig. 1:**
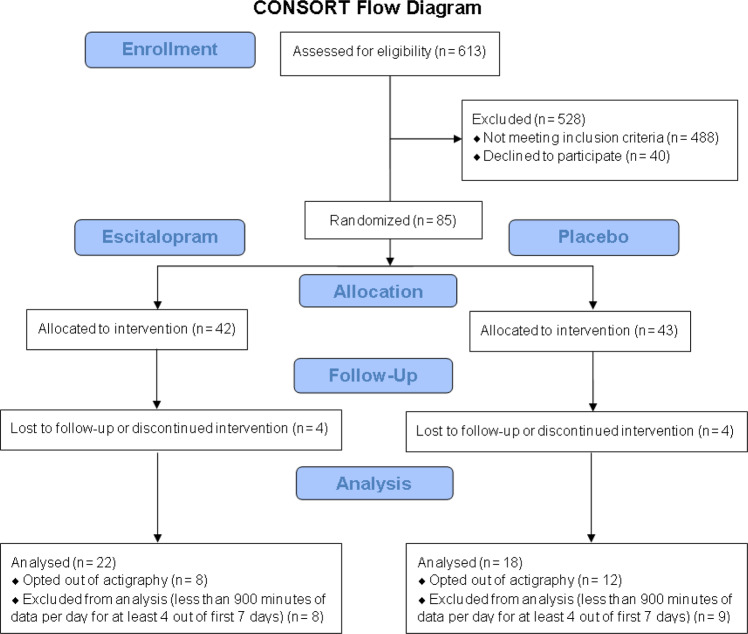
CONSORT flow diagram depicting the progression of participants through the actigraphy study. CONSORT consolidated standards of reporting trials.

The motor activity data in this actigraphy study was acquired as an optional add-on to a randomized, placebo-controlled, double-blind, single-site clinical trial of the SSRI escitalopram with brain imaging (name of the trial registry: Advancing Personalized Antidepressant Treatment Using PET/MRI, registration number: NCT02623205, date of registration: 12/07/2015, https://clinicaltrials.gov/ct2/show/NCT02623205), as described in a previous publication^65^. The Institutional Review Board of Stony Brook University (IRB #570152) approved this study. Written informed consent was obtained from all participants prior to study for participation in this research involving experimentation with human subjects. The CONSORT reporting guidelines were used for reporting^66^. A sample size calculation was performed with two-tailed analysis for α = 0.05 to achieve 80% power and detection of true correlation of at least 0.39. Adults meeting the Diagnostic and Statistical Manual of Mental Disorders, fourth edition (DSM-IV) criteria for MDD were recruited by radio, print, and internet advertising starting in March 2015. Initially, all interested individuals went through a phone screening. After determining their eligibility, prospective participants were brought in for a more in-depth, in-person evaluation. 613 individuals completed the initial phone screening, 488 of whom did not meet the eligibility criteria described below, and 40 chose not to enroll (Fig. 1).

### Eligibility criteria

To be eligible for this study, participants had to have a current diagnosis of MDD and a minimum score of 22 on the Montgomery-Åsberg Depression Rating Scale (MADRS)^67^. The study included participants who were medication-free for 21 days prior to data collection. All participants were provided with the necessary information to make informed decisions. Individuals who lack the capacity to consent were excluded. Further exclusion criteria included significant medical conditions, active physical illness, current efficacious treatment with antidepressants, contraindications to escitalopram including previous failure of escitalopram, electroconvulsive therapy (ECT) within the past 6 months, lifetime history of psychosis or bipolar disorder, active suicidality, high potential for substance use during the study period, significant neurological deficits or previous head injury involving loss of consciousness or motor deficits, or dementia (clinical and neurocognitive criteria).

### Treatment randomization

The participants were washed off any psychotropic medication for three weeks before initiating treatment. Randomization of treatment was performed using a double-blind design in a 1:1 ratio to provide participants with either the first-line treatment with an SSRI^26–28^ called escitalopram (ESC) or placebo. A pseudo-random allocation scheme was generated by the study pharmacist using the computer software, Research Randomizer^®^, before labeling and distributing the medication bottles to study participants. This allowed for blinding of the research participants, study clinicians, raters, and study coordinators. The participants randomized to escitalopram received 10 mg (one pill) for one week, 20 mg (two pills) for two weeks, and 30 mg (three pills) thereafter, as a single daily dose. The increase in dosage depended on clinical judgment based on the participant’s tolerance and response. By the end of the trial, all participants on escitalopram received the maximum recommended dose of 30 mg. Participants on placebo followed the same schedule for the number of placebo pills. The placebo group was included to examine if the actigraphy variables predicted antidepressant response from SSRI or in general regardless of treatment assignment of SSRI vs. placebo. This is because motor activity may change differently due to SSRI as compared to placebo. The depression severity of the participants was quantified by trained raters using the 17-item Hamilton Depression Rating Scale (HDRS) before initiating treatment, and at the end of the first and eighth week of treatment.

### Actigraphy data acquisition

The CONSORT flow diagram for the actigraphy study is provided in Fig. 1. Briefly, 77 participants completed the trial after 8 discontinued, 57 of whom opted for actigraphy. Participants wore the actigraphy monitor, *ActiCal* (Philips Respironics, Mini Mitter Company Inc., Oregon, USA), a uniaxial accelerometer that senses acceleration in a single vertical plane from body movement^68–73^. Participants were given instructions to wear the device on the non-dominant wrist at all times, except when showering. The device recorded digital signal of time series activity data from wrist actigraphy for 1-minute intervals (epoch) for a continuous period of 1 week after initiating treatment. Therefore, the sample rate of the acceleration data acquisition was activity per minute (min^−1^). Electric signal is generated in the ceramic enclosure of the sensor (piezoelectricity) when the sensor is deformed in response to vibrations from movement/activity^74^. The generated signal is amplified and filtered through a bandpass filter^75^, where any acceleration signal outside of 0.5–3.0 Hz was filtered out to remove artifacts or noise^76^. This analog signal is digitized using an analog to digital converter at 32 Hz sampling frequency. In this way, the sensor detects minute-by-minute acceleration (rate of change of displacement every minute), and activity counts per minute are recorded. 40 participants with a minimum of 4 days (≥5760 min) of actigraphy data (without any missing data) from the first week after non-wear data removal (described below) were included in the study. This is in line with previous actigraphy studies for antidepressants that have used data from at least 3 days^38,47^.

### Actigraphy data processing

The raw accelerometer files were processed using the *Actical* software, which included verification of epoch length: 1 min, device location: wrist, sex, age, and height and weight from physical exams. Afterwards, the standard *Choi* algorithm was applied to remove non-wear data with a 90-minute window of consecutive zero counts^77^. This algorithm allows artifacts defined as 2-minute interval of nonzero counts, that has 30-minute consecutive zero counts upstream or downstream. The segmented periods of wear time and activity were appended, with the timestamps intact. Even though the standard practice is to include data from days with >600 min of data per day^78^, this study required 900 min of data per day (for at least 4 out of 7 days) to extract the variables related to 600 most active minutes (M10) and 300 least active minutes (L5) each day^79^. This design minimized any overlapping between the hours included in M10 and L5, and their difference (relative amplitude) was used in the final model.

### Parametric measures

**Fig. 2:**
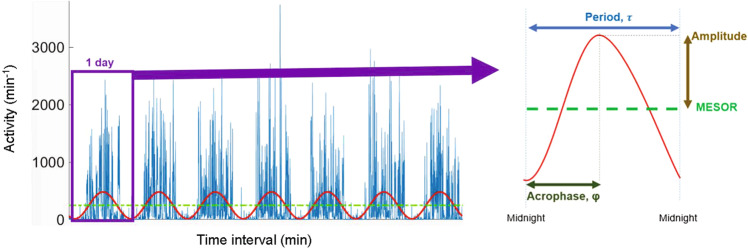
Cosinor fit used to extract parametric measures from continuous actigraphy with minute-by-minute activity. *Cosinor fit* (red line) used to extract parametric measures over the 7 days of continuous actigraphy data (blue lines) from a sample participant with MDD. Each dot on the plot signifies one minute in time and the height of the line indicates motor activity for that minute. 1 day length of data (recorded from midnight to midnight) is indicated by the horizontal length of the purple rectangular box. *Amplitude* (brown double arrow) is indicated at half the distance between peaks of the fitted waveform. *Acrophase* (φ, dark olive, double arrow) refers to the time of the point of peak activity. *MESOR* (Midline Estimating Statistic of Rhythm, green dashed line) indicates the average cycle value.

The preliminary parametric measures included Midline Estimating Statistic of Rhythm (MESOR), amplitude, and acrophase. These parametric measures for the periodic characteristics were obtained using *cosinor analysis* with a least squares approach^80^. This single-component cosinor model used regression to fit a cosine wave to the time series activity data as shown in the equation below (Fig. 2)^81^:

1 $${Y}_{i}\left(t\right)={M}_{i}+{A}_{i}.\cos \left(\frac{2\pi t}{\tau }+{\varphi }_{i}\right)+{e}_{i}(t)$$

In Eq. (1), ${Y}_{i}\left(t\right)$ refers to the motor activity at time $t$ for participant $i$. ${M}_{i}$ indicates the rhythm-adjusted average activity count, known as the Midline Estimating Statistic of Rhythm (MESOR) (Fig. 2, green line). ${A}_{i}$ refers to the amplitude measuring the average difference between the maximum and minimum motor activity, which is half the distance of the peak of the fitted waveform (Fig. 2, brown double arrow). The normalized value of amplitude is circadian quotient (CQ: amplitude/MESOR) that allows comparison of circadian rhythm robustness among participants with varying activity levels by considering both of their daytime and nighttime activity^36^.

$\tau$ indicates the period of 24 h, the assumed duration for one cycle. Acrophase, ${\varphi }_{i},$ measures the time of the peak motor activity, the time of overall highest amplitude, recurring in each cycle (Fig. 2, dark olive double arrow). *e*_*i*_(*t*) is the error term. With an assumed period of *τ* = 24 h, the non-linear problem of fitting a cosine function (Eq. 1) is reduced to a problem with linear parameters. The cosinor analysis is applied using a cycle length of 2π. Using the trigonometric angle sum identity:

$${A}_{i}.\cos \left(\frac{2\pi t}{\tau }+{\varphi }_{i}\right)={A}_{i}.\cos \left({\varphi }_{i}+\frac{2\pi t}{\tau }\right)={A}_{i}.\cos \left({\varphi }_{i}\right)* {A}_{i}.\cos \left(\frac{2\pi t}{\tau }\right)-{A}_{i}.\sin \left({\varphi }_{i}\right)* {A}_{i}.\sin \left(\frac{2\pi t}{\tau }\right)$$

The model in Eq. 1 can be transformed to the model below:

$${Y}_{i}\left(t\right)={M}_{i}+\beta x+\gamma z+{e}_{i}\left(t\right),$$

where $$\beta ={A}_{i}.\cos \left({\phi }_{i}\right){\rm{;}}x=\cos \left(\frac{2\pi t}{\tau }\right){\rm{;}}\gamma ={\rm{ \mbox{-} }}{A}_{i}.\sin \left({\phi }_{i}\right){\rm{;}}$$

$$\,z=\sin \left(\frac{2\pi t}{\tau }\right)$$

### Nonparametric measures

The nonparametric measures include average activity (min^−1^) over the most active 10 consecutive hours (M10) and the least active 5 consecutive hours (L5), relative amplitude (RA), intradaily variability (IV), and interdaily stability (IS). RA reflects the difference between M10 and L5 activity calculated as shown in Eq. (2) below:

2 $${RA}=\frac{M10-L5}{M10+L5}$$

The RA value ranges from 0 to 1, with a high value indicating sufficient balance of rest-active status, whereas a low value indicates insufficient balance of rest-active status. In motor activity research, M10 refers to “daytime activity” in healthy individuals. However, the time of the day of most active hours may vary for an individual with MDD. Therefore, this article refers to M10 as “most active hours.” L5 measures the average activity of five consecutive least active hours (including sleep), known as “nocturnal activity” in a healthy individual, and is referred to as the “least active hours” in this article^43^.

Intradaily variability (IV) estimates the circadian rhythm fragmentation by taking into account the extent and frequency of rest-activity transitions every hour. IV value ranges from 0 to 2, with a higher value indicating a more fragmented rest-activity rhythm due to daytime rest/nappings and/or nighttime arousals^43,82^. An IV value near 0 indicates a strong rest-activity rhythm, while Gaussian noise would generate an IV near 2. IV is obtained by calculating the ratio of the mean squared first derivative of the data and the variance, as shown in Eq. (3) below^82,83^.

3 $${IV}=\frac{N\mathop{\sum }\nolimits_{i=2}^{N}{\left({x}_{i}-{x}_{i-1}\right)}^{2}}{\left(N-1\right)\mathop{\sum }\nolimits_{i=1}^{N}{\left({x}_{i}-\bar{x}\right)}^{2}}$$

Where *N* is the total number of sampling points in the time series data. $\bar{x}$ is the average of all data and ${x}_{i}$ is the individual data point, which is calculated as an hourly value for this equation^83^.

Interdaily stability (IS) estimates the consistency/stability of activity patterns over multiple days and its synchronization to the 24-hour light-dark cycle. IS value ranges from 0 to 1, with a high value indicating stable rhythm with good synchronization^43^.

4 $${IS}=\frac{N\mathop{\sum }\nolimits_{h=1}^{s}{\left({\bar{x}}_{h}-\bar{x}\right)}^{2}}{s\mathop{\sum }\nolimits_{i=1}^{N}{\left({x}_{i}-\bar{x}\right)}^{2}}$$

In Eq. (4), N is the total number of sampling points in the time series data; *s* is the number of sampling points per day. ${\bar{x}}_{h}$ is the hourly mean. $\bar{x}$ is the average of all data and ${x}_{i}$ is the individual data point, which is calculated as an hourly value for this equation^83^.

### Statistical analysis

Each circadian rhythm measure was visualized and summarized (Supplementary Figs. 1–4). For any detected outlier, the associated actigraphy data was visually inspected for unusual patterns, that did not result in the removal of any outliers. All analysis was performed with and without the outliers to test for robustness. The Hamilton Depression Rating Scale (HDRS) scores were positively skewed. Therefore, they were log-transformed to make their distribution and the distribution in change in depression better approximate normal distribution. The distribution of these log-transformed values was subsequently tested for normality using the Kolmogorov-Smirnov test. The change in depression after one week of treatment was calculated as: ΔHDRS1 = ln(Week1-HDRS/Week0-HDRS) = ln(Week1-HDRS) – ln(Week0-HDRS)]. The change in depression after eight weeks of treatment was calculated as: [ΔHDRS8 = ln(Week8-HDRS/Week0-HDRS) = ln(Week8-HDRS) – ln(Week0-HDRS)]. For instance, ΔHDRS1 = −0.176 implies that ratio of Week1-HDRS to Week0-HDRS is ~exp(−0.176) = 0.838, corresponding to 0.838–1.000 = −0.162 or a 16.2% decrease in depressive symptoms at week 1 from baseline. The same method was used for interpreting the values of ΔHDRS8. The parametric measures (CQ and acrophase) and the non-parametric measures (RA, IV, and IS) were standardized before being used in statistical analysis. The binary variables (treatment assignment: SSRI vs. placebo and sex: female vs. male) were analyzed using the Fisher’s exact test. The numeric data was analyzed using two-tailed t-tests and Mann-Whitney U (MW-U a.k.a. Wilcoxon Rank Sum) test. Variable extraction from cosinor analysis and data visualization were performed using *Matlab R2020a* (MathWorks, Natick, MA) and *Python 3.9.0* (Python Software Foundation, Beaverton, OR). Statistical analyses were performed using *R 4.1.0* (RStudio, Boston, MA), *SAS 9.4* (SAS Institute, Cary, NC) and *STATA/SE 13.0* (StataCorp LLC, College Station, TX).

### Relationship of week 1 actigraphy measures with change in depression at week 1 and week 8

Spearman’s correlation was assessed between each of the parametric and nonparametric measures extracted from the first week of treatment and the change in depression after one week [ΔHDRS1 = ln(Week1-HDRS/Week0-HDRS) = ln(Week1-HDRS) – ln(Week0-HDRS)] and eight weeks of treatment [ΔHDRS8 = ln(Week8-HDRS/ Week0-HDRS) = ln(Week8-HDRS) – ln(Week0-HDRS)]. Repeated measures analysis of variance (RM-ANOVA) was performed using a linear mixed model with restricted maximum likelihood (REML) estimation for the repeated measures of treatment week (Week0 vs. Week1). The standardized values of the parametric (CQ and acrophase) and nonparametric (RA, IV, and IS) measures extracted from the first week of treatment were included as fixed effects. Association of these parametric and nonparametric measures with the change in depression from pretreatment values after the first week of treatment [ΔHDRS1 = ln(Week1-HDRS/Week0-HDRS) = ln(Week1-HDRS) – ln(Week0-HDRS)] was the dependent variable in two separate models. Similarly, association of these parametric and nonparametric measures with the change in depression from pretreatment values after the eighth week [ΔHDRS8 = ln(Week8-HDRS/ Week0-HDRS) = ln(Week8-HDRS) – ln(Week0-HDRS)] were analyzed by using ΔHDRS8 as dependent variable in two separate models. All RM-ANOVA models included *n* = 40 participants (random effect) with 1 observation at each of the 2 time points (Week 0 vs. Week 1) for a total of 80 observations. They also included the fixed effects of treatment assignment (SSRI vs. placebo), sex (female vs. male), age, and squared value of age (to address a potential nonlinear relationship with depression)^84^ and their interactions with treatment week.

### Prediction of remitters and non-remitters

The standardized parametric and nonparametric measures were independent variables in two separate multivariate logistic regression models, along with covariates of treatment assignment (SSRI vs. placebo), sex (female vs. male), age and squared value of age. The binary outcome was remitter (free of depression, vs. non-remitter) designated a priori as at least 50% reduction in depression, which is a score of ≤7 on the 17-item Hamilton Depression Rating Scale (HDRS), after eight weeks of treatment^85–87^.

In addition, the most significant predictor from previous RM-ANOVA models was used as an independent variable in a separate logistic regression model. The optimal threshold for identifying positive outcome was designated by calculating the difference between the True Positive Rate and False Positive Rate from the Receiver Operating Characteristic (ROC) curve. The model performance metrics of sensitivity, specificity, positive predictive value and negative predictive value were calculated afterwards after setting the optimal threshold for positive outcome.

### Reporting summary

Further information on research design is available in the Nature Research Reporting Summary linked to this article.

## Supplementary information

Supplementary Information

Reporting Summary

## Data Availability

The authors confirm that all relevant data are available from the authors and can be provided on request.

## Code availability

Standard software applications have been used for data analysis and are mentioned in the methodology.
