# Supplementary material for: Circadian rhythm biomarker from wearable device data is related to concurrent antidepressant treatment response
Source: NPJ Digit Med. 2023 Apr 29;6:81. doi: 10.1038/s41746-023-00827-6 (PMC10148831; doi:10.1038/s41746-023-00827-6)
Supplement: Supplementary file 1 — Supplementary Information [file 41746_2023_827_MOESM1_ESM.pdf]

Visualization and summary statistics of circadian rhythm measures

Variables	Treatment Assignment: Selective Serotonin Reuptake Inhibitor (SSRI) (N=22, 55%)			Placebo (N=18, 45%)			Fisher's exact test	t-test (DF = 38)	MW-U test
	N (%)	Mean (SD)	Median (IQR)	N (%)	Mean (SD)	Median (IQR)	(*P*)	t (*P*)	z (*P*)
**Demographic**									
Sex: F (vs. M)	15 (68)			13 (72)			1.00		
Age (years)		33.74 (16.76)	25.05 (29.60)		27.68 (13.22)	22.70 (7.20)		-1.25 (0.22)	-0.84 (0.40)
**Clinical**									
Week0-HDRS		19.45 (5.20)	19.00 (5.00)		15.83 (3.62)	15.50 (3.00)		**-2.50 (0.02*)**	**-2.74 (0.01*)**
Week1-HDRS		15.59 (4.18)	16.00 (7.00)		14.17 (3.63)	14.00 (3.00)		-1.14 (0.26)	-1.25 (0.21)
Week8-HDRS		11.91 (6.89)	10.00 (8.00)		8.69 (5.57)	7.00 (8.00)		-1.60 (0.12)	-1.47 (0.14)
ΔHDRS1		-0.22 (0.16)	-0.25 (0.17)		-0.12 (0.30)	-0.18 (0.32)		1.43 (0.16)	1.33 (0.18)
ΔHDRS8		-0.64 (0.54)	-0.56 (0.76)		-0.89 (0.93)	-0.68 (0.93)		-1.08 (0.29)	-0.64 (0.52)

Supplementary Table 1 Participants' demographics and clinical characteristics stratified by treatment assignment. *N*: sample size. DF: degrees of freedom = 38 for all the variables in the t-test. *MW-U test*: Mann-Whitney U test. *SD*: standard deviation. *IQR*: interquartile range (75th percentile – 25th percentile). *F*: Female. *M*: Male. *HDRS*: Hamilton Depression Rating Scale. *Week0-HDRS*: Hamilton Depression Rating Scale score at baseline. *Week1-HDRS*: Hamilton Depression Rating Scale score after first week of treatment. *Week8-HDRS*: Hamilton Depression Rating Scale score after eight weeks of treatment. *ΔHDRS1*: change in depression severity after first week of treatment, ln(Week1-HDRS/ Week0-HDRS), with a higher value indicating more depression symptoms and a lower value indicating improvement in depression. *ΔHDRS8*: change in depression severity after eight weeks of treatment, ln(Week8-HDRS/ Week0-HDRS), with a higher value indicating more depression symptoms and a lower value indicating improvement in depression. The *P*-values that are statistically significant are indicated in bold for $^*P < 0.05$ at $\alpha = 0.05$.

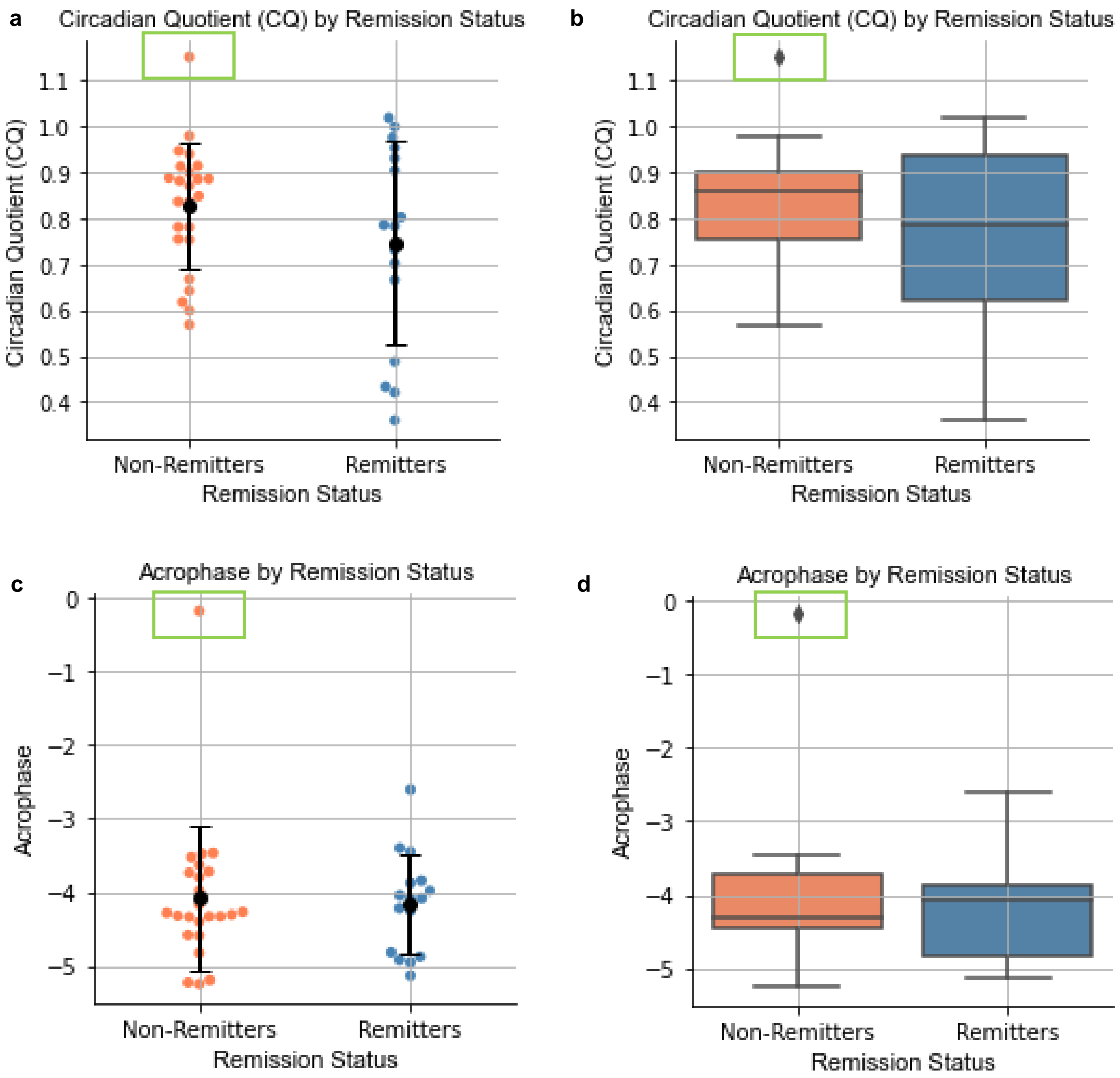
a
Circadian Quotient (CQ) by Remission Status
Circadian Quotient (CQ)
1.1
1.0
0.9
0.8
0.7
0.6
0.5
0.4
Non-Remitters
Remitters
Remission Status
b
Circadian Quotient (CQ) by Remission Status
Circadian Quotient (CQ)
1.1
1.0
0.9
0.8
0.7
0.6
0.5
0.4
Non-Remitters
Remitters
Remission Status
c
Acrophase by Remission Status
Acrophase
0
−1
−2
−3
−4
−5
Non-Remitters
Remitters
Remission Status
d
Acrophase by Remission Status
Acrophase
0
−1
−2
−3
−4
−5
Non-Remitters
Remitters
Remission Status

Supplementary Figure 1: Parametric measures of circadian rhythm by remission status. Mean (black circle) and standard deviation (black error bars) are reported for comparing non-remitters and remitters for the measures of **a** circadian quotient (CQ) and **c** acrophase. In addition, the median (center line) and interquartile range (box) with 25th and 75th percentiles (bounds of box) are reported for comparing non-remitters and remitters for the measures of **b** circadian quotient (CQ) and **d** acrophase. The whiskers go from 25th percentile to the minimum value and 75th percentile to the maximum value. *Non-Remitters* (orange)**:** Week8-HDRS > 7. *Remitters* (blue): Week8-HDRS ≤ 7. *HDRS*: Hamilton Depression Rating Scale. *Week8-HDRS*: Hamilton Depression Rating Scale score after eight weeks of treatment. The actigraphy data associated with the two outliers marked inside the green rectangle for CQ = 1.15 and acrophase = - 0.18 were visually inspected to assess for any unusual patterns that did not lead to removal of any data. All analysis was performed with and without the outliers to test for robustness. The *P*-values that are statistically significant are indicated in bold for **P* < 0.05 at α = 0.05.

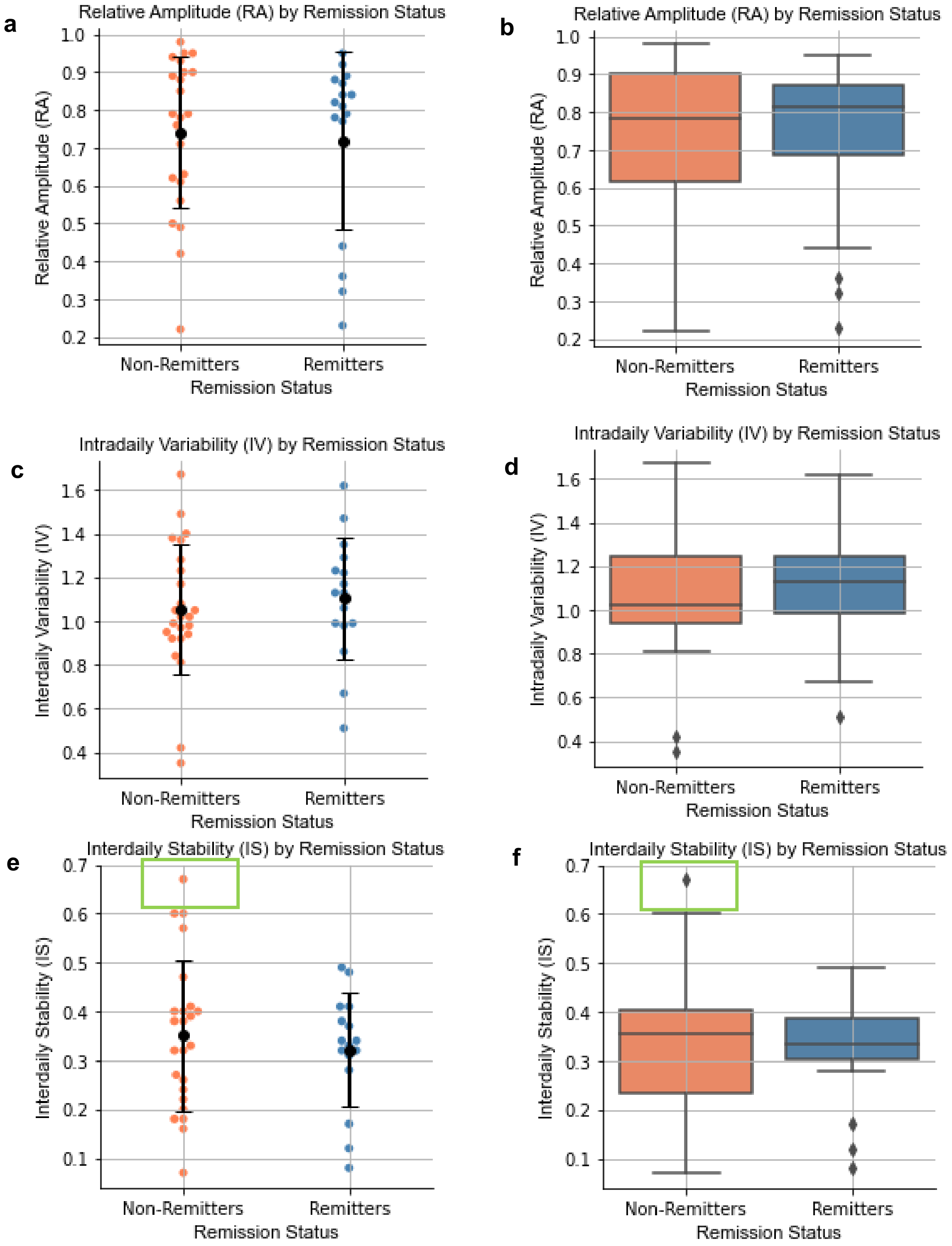
a
Relative Amplitude (RA) by Remission Status
Relative Amplitude (RA)
Non-Remitters
Remitters
Remission Status
b
Relative Amplitude (RA) by Remission Status
Relative Amplitude (RA)
Non-Remitters
Remitters
Remission Status
c
Intradaily Variability (IV) by Remission Status
Interdaily Variability (IV)
Non-Remitters
Remitters
Remission Status
d
Intradaily Variability (IV) by Remission Status
Intradaily Variability (IV)
Non-Remitters
Remitters
Remission Status
e
Interdaily Stability (IS) by Remission Status
Interdaily Stability (IS)
Non-Remitters
Remitters
Remission Status
f
Interdaily Stability (IS) by Remission Status
Interdaily Stability (IS)
Non-Remitters
Remitters
Remission Status

Supplementary Figure 2: Nonparametric measures of circadian rhythm by remission status. Mean (black circle) and standard deviation (black error bars) are reported for comparing non-remitters and remitters for the measures of **a** relative amplitude (RA), **c** intradaily variability (IV) and **e** interdaily stability (IS). In addition, the median (center line) and interquartile range (box) with 25th and 75th percentiles (bounds of box) are reported for comparing non-remitters and remitters for the measures of **b** relative amplitude (RA), **d** intradaily variability (IV) and **f** interdaily stability (IS). The whiskers go from 25th percentile to the minimum value and 75th percentile to the maximum value. *Non-Remitters* (orange)**:** Week8-HDRS > 7. *Remitters* (blue): Week8-HDRS ≤ 7. *HDRS*: Hamilton Depression Rating Scale. *Week8-HDRS*: Hamilton Depression Rating Scale score after eight weeks of treatment. The actigraphy data associated with the outlier marked inside the green rectangle for IS = 0.67 was visually inspected to assess for any unusual patterns that did not lead to removal of any data. All analysis was performed with and without the outlier to test for robustness. The *P*-values that are statistically significant are indicated in bold for *$P < 0.05$ at $\alpha = 0.05$.

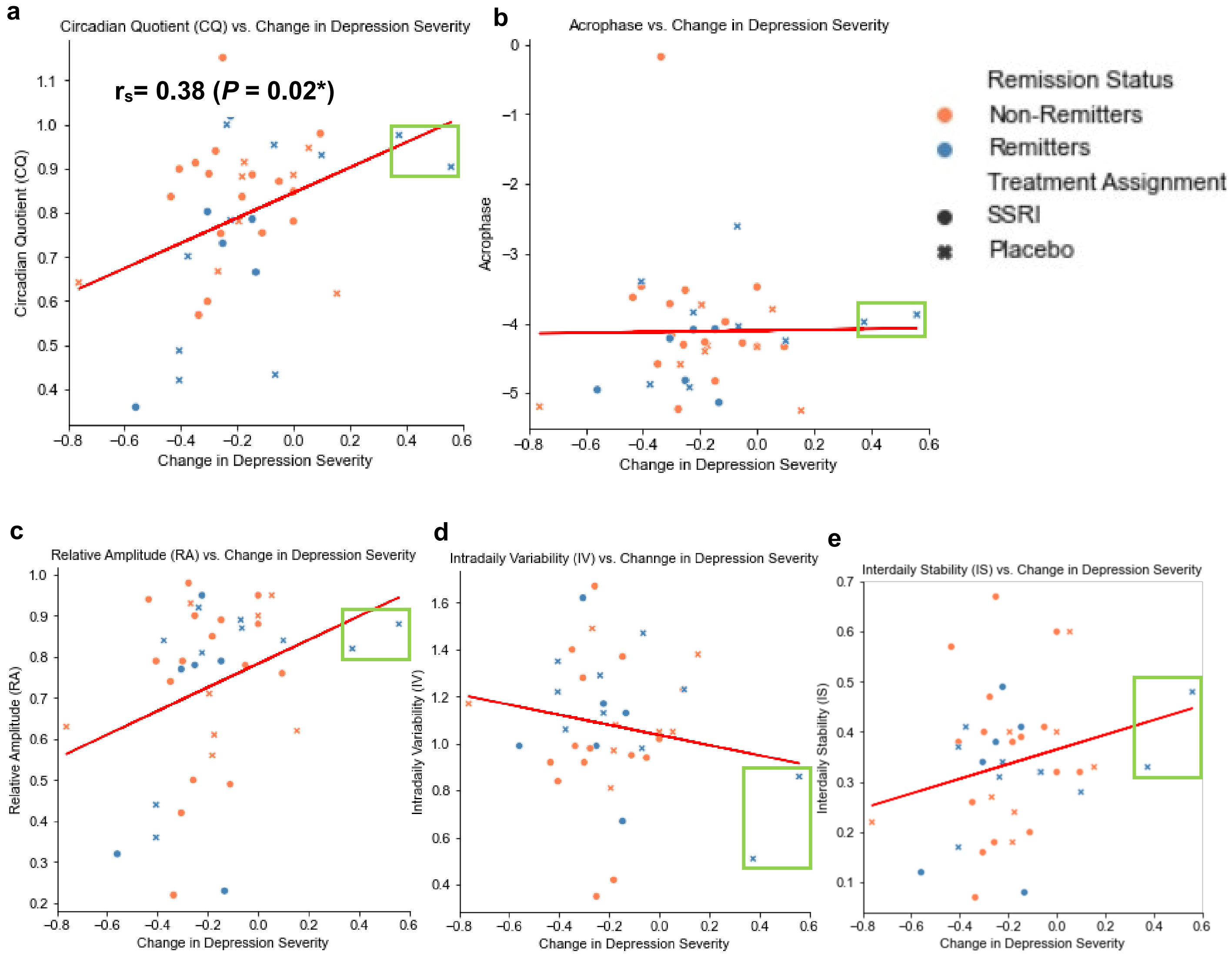

Supplementary Figure 3: Parametric and nonparametric measures of circadian rhythm and their association with change in depression after first week of antidepressant treatment. The relationship between change in depression severity after first week of antidepressant treatment and the parametric measures including **a** circadian quotient (CQ) and **b** acrophase and the nonparametric measures including the **c** relative amplitude (RA), **d** intradaily variability (IV), and **e** interdaily stability (IS). The red line indicates the regression line for the circadian rhythm measures over change in depression severity after first week of antidepressant treatment (ΔHDRS1). *ΔHDRS1*: change in depression severity after first week of treatment, ln(Week1-HDRS/ Week0-HDRS), with a higher value indicating more depression symptoms and a lower value indicating improvement in depression. *HDRS*: Hamilton Depression Rating Scale. *Week1-HDRS*: Hamilton Depression Rating Scale score after first week of treatment. *Week0-HDRS*: Hamilton Depression Rating Scale score at baseline. r_s: Spearman's correlation coefficient, which was used to assess correlation between each of the parametric and nonparametric measures extracted from the first week of treatment and the change in depression after one week (ΔHDRS1). The two extreme values (ΔHDRS1 = 0.37 and 0.56) marked inside the green rectangle were removed to assess for the robustness of the findings on the relationship between the circadian rhythm measures and depression. All analysis was performed with and without the outliers to test for robustness. The *P*-values that are statistically significant are indicated in bold for $^*P < 0.05$ at $\alpha = 0.05$.

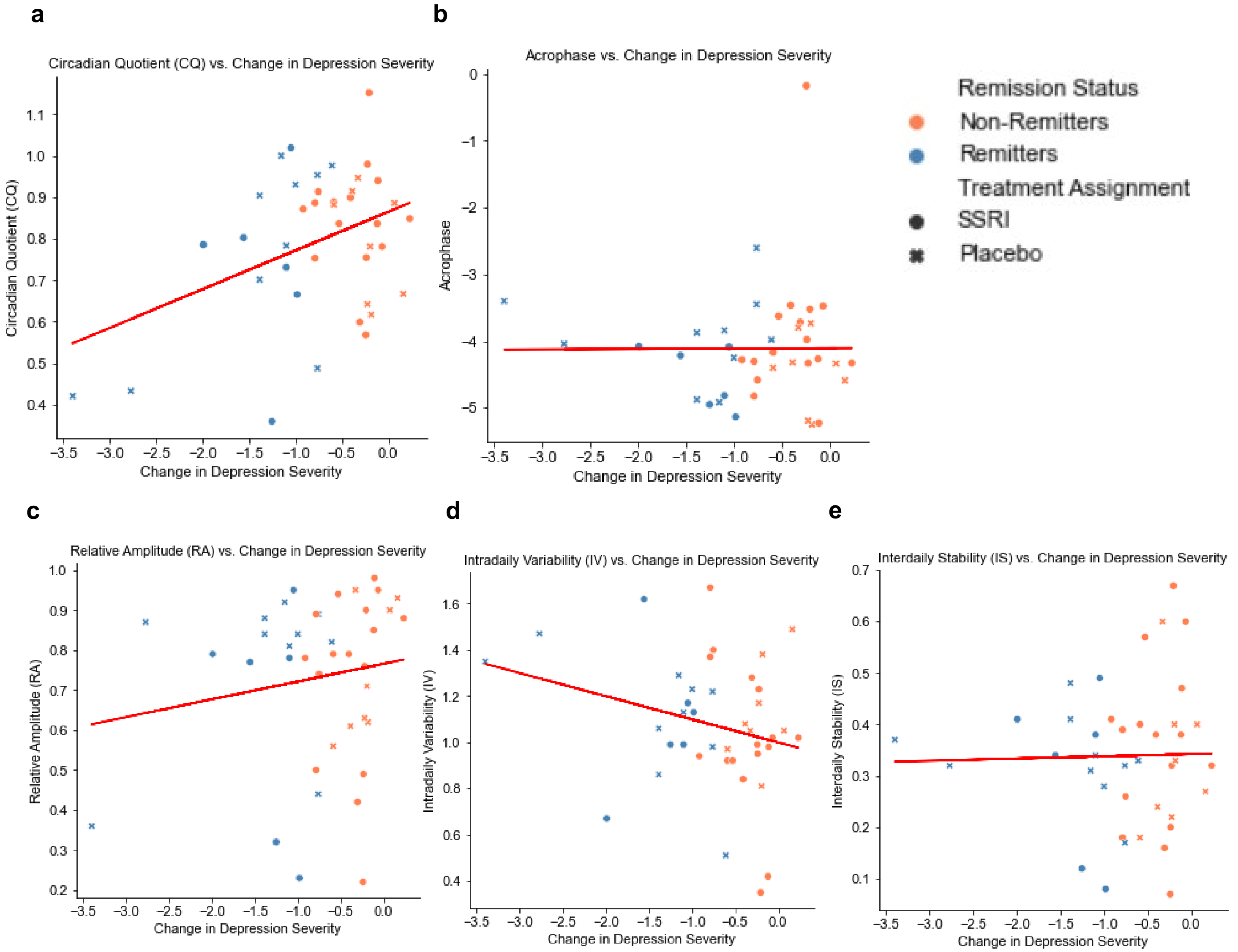

Supplementary Figure 4: Parametric and nonparametric measures of circadian rhythm and their association with change in depression after eight weeks of antidepressant treatment. The relationship between change in depression severity after first week of antidepressant treatment and **a** circadian quotient (CQ), **b** acrophase, **c** relative amplitude (RA), **d** intradaily variability (IV), and **e** interdaily stability (IS). The red line indicates the regression line for the circadian rhythm measures over change in depression severity after eight weeks of antidepressant treatment (ΔHDRS8). *ΔHDRS8*: change in depression severity after eight weeks of treatment, ln(Week8-HDRS/ Week0-HDRS), with a higher value indicating more depression symptoms and a lower value indicating improvement in depression. *HDRS*: Hamilton Depression Rating Scale. *Week8-HDRS*: Hamilton Depression Rating Scale score after eight weeks of treatment. *Week0-HDRS*: Hamilton Depression Rating Scale score at baseline. The *P*-values that are statistically significant are indicated in bold for $^*P < 0.05$ at $\alpha = 0.05$.
